# Global, regional, and national burden trends of spinal fractures from 1990 to 2021: a population-based study

**DOI:** 10.1097/JS9.0000000000003290

**Published:** 2025-10-06

**Authors:** Zhizhong Shang, Yubao Lu, Jiale He, Jiawei Di, Zifang Huang, Pan Zhou, Lang Mai, Ruijue Zhu, Yankui Liu, Mao Pang, Lei He, Bin Liu, Limin Rong

**Affiliations:** a Department of Spine Surgery, The Third Affiliated Hospital, Sun Yat-Sen University, Guangzhou, Guangdong, China; b Guangdong Provincial Center for Engineering and Technology Research of Minimally Invasive Spine Surgery, Guangzhou, Guangdong, China; c Guangdong Provincial Center for Quality Control of Minimally Invasive Spine Surgery, Guangzhou, Guangdong, China; d Spine and Spinal Cord Injury Treatment Center of The Third Affiliated Hospital, Sun Yat-Sen University, Guangzhou, Guangdong, China; e Scoliosis Center, The Third Affiliated Hospital, Sun Yat-Sen University, Guangzhou, Guangdong, China

**Keywords:** disease burden, elderly, public health, spinal fractures, trend changes

## Abstract

**Background:**

Spinal fractures have become a significant cause of health loss and an increasing disease burden due to their high incidence and disability risk. However, the current understanding of the disease burden and its trend remains limited, which hinders the effectiveness of public health interventions.

**Methods:**

Data for this study were sourced from the standardized Global Burden of Disease (GBD) 2021 database. Various analytical methods, including trend analysis, correlation analysis, age-period-cohort analysis, frontier analysis, health inequality analysis, and predictive analysis, were used to explore the current status and trends of the disease burden associated with spinal fractures.

**Results:**

From 1990 to 2021, the age-standardized rate (ASR) of spinal fractures globally showed a downward trend, and this trend is expected to continue in the future. However, the absolute number of spinal fracture cases has steadily increased. Since 2018, the rate of decline in the age-standardized prevalence rate and years lived with disability has slowed. In 2021, there were 7.4975 million new cases of spinal fractures globally, with 5.3714 million existing patients, resulting in 546 000 years lived with disability. Significant disease burden inequalities were observed across different regions, with a notable increase in the burden as the Socio-Demographic Index (SDI) rises. High-SDI regions and countries faced the most severe disease burden. The ASR remained low for individuals under 60 years of age, with both the ASR and actual cases higher in males than females. For individuals aged 60 years and above, the ASR significantly increased, with females surpassing males. The ASR shows an overall decreasing trend across different genders and age groups, but the absolute inequality is greater in men than in women. Additionally, falls, other exposures to mechanical forces, and motor vehicle road injuries were the leading causes of spinal fractures.

**Conclusion:**

Although the ASR of spinal fractures has significantly decreased, the absolute number of patients continues to rise, and heterogeneity exists across countries, regions, age groups, genders, and over time. These findings highlight the need for more targeted and personalized public health interventions in the future.

## Background

Spinal fractures, resulting from either direct or indirect external forces, cause compression deformation or fracture of the vertebral body, accounting for approximately 5–6% of all fractures^[1]^. These injuries are often accompanied by damage to nerves and blood vessels, leading to chronic pain, significant reductions in quality of life, and even premature death^[2]^. Analysis of 71 051 patients with thoracolumbar fractures reveals that approximately 15.81% of these patients have concurrent spinal cord injury (SCI)^[3]^. In the United States, the overall prevalence of spinal fractures is estimated to be 5.4%, with a significant increase in prevalence as age rises^[4]^. Osteoporotic spinal fractures, closely linked to aging, often result in severe back pain, functional impairment, respiratory complications, and significantly increased mortality^[5,6]^. Surgical treatment is the primary approach for managing spinal fractures; however, its complication rate can be as high as 15%, with a strong association with the occurrence of major bleeding, severe infections, and other complications^[7]^.

With substantial advancements in lifestyle, medical care, economic development, and policy changes, it is imperative to reassess the actual disease burden of spinal fractures. Accurate quantification of disease burden lays the foundation for implementing effective preventive measures and early interventions, thereby reducing the public health burden associated with spinal fractures^[8]^. Although several studies have previously assessed the prevalence of spinal fractures, most of these studies are limited to specific countries, such as the United States^[9]^, China^[10,11]^, Japan^[12]^, Sweden^[12]^, and South Korea^[5]^, and often suffer from small sample sizes, limited timeframes, and are typically outdated, single-center studies. Dong *et al*’s study provides an initial overview of the global and national disease burden of spinal fractures but fails to explore in depth the geographical distribution, age-gender-time patterns, and the relationship with the Socio-Demographic Index (SDI), nor does it predict future trends^[13]^. This limits the utility of the study for providing actionable public health policy recommendations. Moreover, the data based on GBD 2019 are no longer sufficient to offer reliable evidence for current global public health practices. Therefore, this study utilizes the standardized GBD 2021 database to conduct a comprehensive analysis of the global and national disease burden of spinal fractures over the past 32 years. It includes trends in age, gender, and time, as well as disease etiology, future developments, and health inequalities. This research will provide a solid epidemiological foundation for the prevention, early diagnosis, and treatment of spinal fractures, as well as global public health responses.

## Materials and methods

### Data source and definitions

The data used in this study were obtained from the Global Burden of Disease Study 2021 (GBD 2021) database (https://vizhub.healthdata.org). GBD 2021 systematically integrates 328 938 data sources worldwide, including vital registration systems, hospital records, outpatient surveys, epidemiological studies, surveillance systems, and published literature, to construct a standardized framework for disease burden estimation. The dataset covers 204 countries and 811 subnational locations. For regions not directly represented – such as remote areas in some low-income countries – data gaps are filled using the Bayesian meta-regression tool DisMod-MR 2.1. This model incorporates geospatial covariates, temporal trends, and population age-sex structures to generate statistically optimal estimates. All results are reported with 95% uncertainty intervals (UIs) to reflect variability in the data and model uncertainty^[14]^. In GBD 2021, spinal fractures are classified according to the International Statistical Classification of Diseases, 10th Revision (ICD-10), using codes S12.0–S12.9, S22.0–S22.1, S32.0–S32.7, and T08^[14]^. The injury estimation framework of GBD 2021 emphasizes attribution of each injury to an external cause. Notably, GBD does not explicitly distinguish between traumatic and pathological fractures (e.g., osteoporotic fractures). Instead, all cases meeting the above ICD coding criteria are attributed to a specific injury mechanism based on recorded or inferred external causes of injury^[14]^.

**HIGHLIGHTS**

- The global burden of spinal fractures declined from 1990 to 2021, although the rate of decline has slowed since 2018.
- Age-standardized rates significantly increased in those aged 60 years and above, with females surpassing males.
- High-SDI regions experienced the greatest burden, highlighting the impact of socioeconomic factors.
- Falls, mechanical exposure, and traffic accidents are the primary causes of spinal fractures.
- Despite the global decline, more targeted public health interventions are needed in high-burden and aging populations.

For this study, we extracted data on age-standardized incidence rates (ASIR), prevalence rates (ASPR), and years lived with disability (ASYR), along with their corresponding 95% UIs, for spinal fractures from 1990 to 2021. Data were stratified by geographical location (global, 5 SDI regions, 21 GBD regions, and 204 countries and territories), age (0–95+ years), and sex. The SDI is a composite measure that accounts for factors such as income level, education, and fertility rate to reflect the development status of a country or region. The 204 countries and regions are categorized into 5 SDI groups: high, high-middle, middle, low-middle, and low SDI regions^[14,15]^.

### Statistical methods

A log-linear regression model was employed to assess the temporal trends of age-standardized rate (ASR) by fitting calendar year against the natural logarithm of ASR. The model is specified as: ln(ASR) = *β*₀ + *β*₁ × year + *ε*, where *β*₀ represents the intercept, *β*₁ is the regression coefficient (i.e., the slope) for the year variable, and *ε* denotes the error term. The estimated annual percentage change (EAPC) was calculated using the formula: EAPC = 100 × [exp(*β*₁) − 1]. The 95% confidence interval (95% CI) for the EAPC was derived from the standard error of *β*₁. To further capture changes in temporal trends, Joinpoint regression analysis was applied, with the number and location of joinpoints determined using a Monte Carlo permutation test. The number of permutations was set at 10 000, and the significance level (*α*) was specified as 0.05. Joinpoints with *P* < 0.05 were considered statistically significant. Based on the final model, annual percentage change (APC) and its 95% CI were calculated for each segment, along with the average annual percentage change (AAPC) across the entire study period. An increasing trend was indicated when the 95% CI for EAPC, AAPC, or APC was entirely above zero; a decreasing trend was inferred when the 95% CI was entirely below zero; and a stable trend was defined when the interval included zero^[15,16]^.

To quantify the relationship between SDI and disease burden, this study first employed Spearman rank correlation analysis to explore the strength of the correlation between SDI and ASR, assessing the correlation with correlation coefficients and *P*-values. Based on this, frontier analysis was used to evaluate the optimization potential of the disease burden. Specifically, the optimal practice frontier based on SDI was constructed to calculate the “effective difference” between the observed values and theoretical optimal values for each country or region. This difference represents the gap between the actual disease burden observed and the potential minimum disease burden that could be achieved given the SDI^[15]^. In addition, the slope index of inequality (SII) was used to directly measure the absolute disparity in burden between the highest and lowest ends of the SDI spectrum, highlighting regions most in need of targeted resource allocation. SII is an absolute measure of health inequality derived from a linear regression model. Regions are ranked from lowest to highest according to socioeconomic status, assigned a relative rank score, and then regressed against the health indicator. The resulting slope (SII) reflects the gradient of health outcomes across the socioeconomic scale. Relative inequality was further assessed using the concentration index (CII), calculated by integrating the Lorenz concentration curve over the interval from −1 to 1. CII reflects the extent and direction of inequality in the distribution of disease burden. A positive CII indicates concentration of burden in higher-SDI countries, whereas a negative value implies a disproportionate burden in lower-SDI settings^[15]^.

The study also employed a Poisson distribution to construct age-period-cohort models to analyze the effects of age, period, and cohort on ASR, with parameter significance tested using the Wald χ^2^ test. Based on this, the BAPC and INLA packages in R were used, applying the BAPC model and the integrated nested Laplace approximation algorithm to predict ASR from 2022 to 2050^[15,16]^. All statistical analyses and graphs were performed using R 4.4.2 and Excel 2021. All hypothesis tests were two-tailed, and differences were considered statistically significant when the *P*-value was less than 0.05 or when the 95% CI did not include 0. In addition, the work has been reported in line with the STROCSS criteria^[17]^.

## Results

### Global, regional, and national burden

At the global level, the ASR of spinal fractures showed a significant downward trend from 1990 to 2021 (with EAPCs and their 95% CIs all being negative, as shown in Table 1 and Supplemental Digital Content Figure S1, available at: http://links.lww.com/JS9/F264). The ASIR decreased from 115.75 per 100 000 in 1990 to 92.75 per 100 000 in 2021 (a reduction of 19.87%), while the ASPR fell from 81.55 per 100 000 to 65.19 per 100 000 (a decrease of 20.06%). The ASYR also declined from 8.33 per 100 000 to 6.62 per 100 000 (a reduction of 20.53%; Table 1). However, the absolute number of cases increased progressively from 1990 to 2021. In 2021, there were 7.4975 million new cases of spinal fractures globally (95% UI: 5.8350, 9.7373), comprising 4.2837 million males (95% UI: 3.3896, 5.4201) and 3.2138 million females (95% UI: 2.3806, 4.3987). The total number of existing patients was 5.3714 million (95% UI: 4.7038, 6.1961), with 2.6944 million males (95% UI: 2.3374, 3.0855) and 2.6770 million females (95% UI: 2.2961, 3.0951). The resulting years lived with disability (YLDs) amounted to 546 000 (95% UI: 366 600, 757 100), with 278 300 (95% UI: 189 000, 385 200) YLDs for males and 267 700 (95% UI: 177 500, 370 800) YLDs for females.

**Table 1:** Age-standardized spinal fractures burden results for the global population, 5 SDI regions, and 21 GBD regions

Location	ASIR			ASPR			ASYLDs		
	1990 (per 100 000 population, 95% UI)	2021 (per 100 000 population, 95% UI)	EAPCs (95% CI)	1990 (per 100 000 population, 95% UI)	2021 (per 100 000 population, 95% UI)	EAPCs (95% CI)	1990 (per 100 000 population, 95% UI)	2021 (per 100 000 population, 95% UI)	EAPCs (95% CI)
Global	115.75 (91.28, 145.92)	92.75 (72.12, 119.99)	−0.77 (−0.81, –0.72)	81.55 (71.55, 93.04)	65.19 (56.89, 75.28)	−0.79 (−0.83, –0.75)	8.33 (5.61, 11.49)	6.62 (4.43, 9.20)	−0.81 (−0.85, –0.77)
SDI									
High SDI	190.52 (147.68, 245.09)	157.17 (119.04, 207.76)	−0.68 (−0.70, –0.65)	156.10 (137.89, 174.99)	131.65 (114.84, 149.24)	−0.61 (−0.63, –0.59)	16.00 (10.84, 22.04)	13.36 (8.95, 18.51)	−0.64 (−0.66, –0.62)
High-middle SDI	135.40 (105.36, 171.80)	109.16 (83.64, 142.31)	−0.89 (−0.97, –0.81)	83.07 (72.53, 95.12)	64.26 (55.80, 74.54)	−1.01 (−1.10, –0.92)	8.56 (5.75, 11.85)	6.63 (4.46, 9.32)	−1.00 (−1.09, –0.91)
Middle SDI	83.76 (66.15, 105.83)	76.23 (58.96, 99.58)	−0.23 (−0.30, –0.16)	39.70 (33.66, 46.41)	39.57 (33.59, 46.45)	−0.05 (−0.15, 0.04)	4.14 (2.78, 5.85)	4.09 (2.76, 5.74)	−0.08 (−0.17, 0.01)
Low-middle SDI	80.93 (64.61, 103.72)	69.74 (54.83, 90.51)	−0.62 (−0.74, –0.49)	38.55 (32.22, 45.39)	36.65 (31.13, 42.92)	−0.26 (−0.33, –0.18)	3.98 (2.72, 5.63)	3.76 (2.56, 5.27)	−0.28 (−0.36, –0.21)
Low SDI	74.64 (58.14, 97.29)	63.40 (49.74, 80.95)	−0.41 (−0.64, –0.17)	33.91 (27.73, 43.43)	33.49 (27.67, 41.42)	−0.04 (−0.15, 0.07)	3.52 (2.38, 5.08)	3.44 (2.39, 4.87)	−0.06 (−0.18, 0.05)
GBD 21 regions									
Andean Latin America	86.87 (69.52, 109.34)	73.08 (57.51, 93.44)	−0.38 (−0.49, –0.27)	35.24 (28.42, 43.90)	32.29 (26.87, 39.16)	−0.21 (−0.27, –0.15)	3.74 (2.49, 5.44)	3.41 (2.28, 4.81)	−0.23 (−0.28, –0.17)
Australasia	256.41 (200.62, 325.05)	232.18 (174.33, 303.58)	−0.22 (−0.31, –0.13)	196.89 (174.25, 224.91)	182.32 (158.11, 208.92)	−0.10 (−0.18, –0.01)	20.19 (13.77, 27.95)	18.56 (12.45, 25.78)	−0.13 (−0.21, –0.05)
Caribbean	73.69 (59.04, 92.42)	85.96 (68.43, 109.85)	0.43 (−0.36, 1.21)	34.05 (28.69, 40.37)	42.64 (36.12, 50.54)	0.81 (0.37, 1.25)	3.58 (2.37, 5.08)	4.42 (3.04, 6.15)	0.76 (0.30, 1.23)
Central Asia	112.43 (89.00, 143.51)	90.11 (71.10, 117.03)	−0.90 (−1.10, –0.70)	45.45 (36.96, 55.12)	37.62 (30.74, 45.71)	−0.73 (−0.84, –0.61)	4.84 (3.19, 6.87)	3.98 (2.64, 5.66)	−0.74 (−0.86, –0.63)
Central Europe	215.29 (165.39, 285.84)	163.90 (123.54, 221.11)	−1.06 (−1.13, –0.99)	93.37 (77.43, 113.72)	71.09 (58.61, 87.02)	−1.05 (−1.12, –0.98)	9.73 (6.33, 13.86)	7.44 (4.81, 10.66)	−1.04 (−1.11, –0.97)
Central Latin America	130.24 (101.90, 167.90)	89.37 (69.36, 114.98)	−0.72 (−0.94, –0.49)	58.28 (48.51, 69.95)	40.95 (34.49, 49.06)	−0.77 (−0.94, –0.60)	6.10 (4.09, 8.64)	4.29 (2.90, 6.02)	−0.76 (−0.94, –0.59)
Central Sub-Saharan Africa	53.51 (42.85, 65.95)	47.93 (37.98, 58.52)	−1.18 (−1.91, –0.45)	25.31 (21.06, 30.39)	25.97 (21.33, 32.14)	−0.34 (−0.71, 0.03)	2.63 (1.80, 3.73)	2.69 (1.87, 3.82)	−0.38 (−0.77, 0.01)
East Asia	68.21 (52.45, 88.53)	75.19 (56.50, 100.88)	−0.03 (−0.36, 0.31)	34.80 (29.75, 40.39)	39.83 (33.85, 46.73)	0.17 (−0.12, 0.47)	3.64 (2.42, 5.12)	4.13 (2.73, 5.86)	0.14 (−0.15, 0.43)
Eastern Europe	210.83 (163.25, 274.93)	168.73 (130.90, 221.27)	−1.05 (−1.34, –0.76)	87.28 (71.85, 106.35)	72.13 (59.51, 87.22)	−0.95 (−1.27, –0.64)	9.20 (6.02, 13.16)	7.60 (4.98, 10.83)	−0.96 (−1.27, –0.65)
Eastern Sub-Saharan Africa	83.97 (57.93, 130.52)	46.21 (36.44, 58.42)	−1.50 (−2.00, –0.99)	33.02 (25.13, 45.62)	25.57 (20.56, 33.54)	−0.69 (−0.93, –0.45)	3.47 (2.27, 5.43)	2.64 (1.82, 3.73)	−0.73 (−0.98, –0.48)
High-income Asia Pacific	191.02 (148.76, 244.20)	130.33 (99.13, 170.30)	−1.42 (−1.54, –1.31)	153.14 (136.73, 172.58)	107.14 (95.11, 121.49)	−1.36 (−1.48, –1.24)	15.85 (10.79, 21.83)	11.06 (7.51, 15.20)	−1.37 (−1.48, –1.25)
High-income North America	177.24 (137.54, 223.05)	158.96 (122.41, 206.63)	−0.41 (−0.52, –0.29)	157.26 (139.37, 176.33)	157.60 (138.08, 178.17)	−0.01 (−0.08, 0.07)	16.08 (10.96, 22.25)	15.76 (10.54, 21.65)	−0.08 (−0.16, –0.00)
North Africa and Middle East	104.06 (84.64, 127.44)	98.91 (77.90, 125.93)	0.31 (0.11, 0.50)	46.75 (38.24, 57.96)	46.08 (37.31, 57.72)	0.17 (0.08, 0.25)	4.91 (3.37, 6.95)	4.80 (3.30, 6.87)	0.15 (0.06, 0.25)
Oceania	51.91 (40.94, 65.99)	61.00 (46.98, 79.40)	0.12 (−0.38, 0.61)	25.82 (22.07, 30.06)	31.39 (26.72, 36.65)	0.40 (0.15, 0.66)	2.68 (1.77, 3.79)	3.24 (2.16, 4.53)	0.38 (0.11, 0.64)
South Asia	83.33 (65.19, 110.14)	75.77 (57.31, 101.33)	−0.45 (−0.57, –0.34)	41.55 (34.95, 49.08)	42.04 (35.53, 50.11)	−0.08 (−0.15, –0.02)	4.24 (2.88, 6.01)	4.26 (2.85, 6.00)	−0.11 (−0.18, –0.04)
Southeast Asia	72.45 (58.16, 90.58)	60.26 (47.65, 76.78)	−0.61 (−0.86, –0.35)	34.11 (28.72, 40.69)	31.12 (26.53, 36.58)	−0.32 (−0.44, –0.21)	3.57 (2.42, 5.05)	3.22 (2.22, 4.53)	−0.35 (−0.47, –0.22)
Southern Latin America	153.26 (120.76, 196.29)	149.29 (115.32, 191.82)	−0.06 (−0.19, 0.06)	115.51 (103.12, 129.32)	114.53 (101.87, 129.44)	−0.02 (−0.10, 0.07)	11.95 (8.09, 16.52)	11.82 (7.96, 16.34)	−0.03 (−0.12, 0.06)
Southern Sub-Saharan Africa	71.74 (56.47, 91.52)	52.06 (41.43, 65.02)	−1.20 (−1.33, –1.08)	35.73 (30.27, 41.44)	25.56 (21.80, 29.61)	−1.27 (−1.41, –1.12)	3.75 (2.55, 5.20)	2.66 (1.83, 3.68)	−1.28 (−1.42, –1.14)
Tropical Latin America	135.60 (103.86, 181.36)	106.31 (81.32, 139.24)	−0.73 (−0.87, –0.59)	59.75 (49.65, 71.50)	49.15 (41.40, 58.44)	−0.62 (−0.71, –0.52)	6.25 (4.09, 9.00)	5.12 (3.39, 7.31)	−0.63 (−0.73, –0.53)
Western Europe	221.86 (167.70, 294.33)	177.62 (128.55, 243.91)	−0.76 (−0.82, –0.70)	188.54 (164.92, 212.98)	151.43 (131.99, 172.21)	−0.75 (−0.80, –0.71)	19.29 (13.06, 26.44)	15.45 (10.37, 21.43)	−0.76 (−0.81, –0.72)
Western Sub-Saharan Africa	47.18 (38.12, 59.47)	44.76 (35.81, 56.18)	−0.23 (−0.35, –0.12)	22.07 (18.60, 26.13)	22.60 (19.20, 26.36)	0.02 (−0.04, 0.09)	2.30 (1.55, 3.21)	2.35 (1.60, 3.30)	0.02 (−0.05, 0.08)

Among the five SDI regions, a horizontal comparison revealed that the ASR of spinal fractures in 2021 increased with higher SDI levels. The high SDI region had the highest ASR, with an ASIR of 157.17 per 100 000, an ASPR of 131.65 per 100 000, and ASYR of 13.36 per 100 000. Conversely, the low SDI region exhibited the lowest ASR, with an ASIR of 63.40 per 100 000, an ASPR of 33.49 per 100 000, and ASYR of 3.44 per 100 000. Longitudinal comparisons from 1990 to 2021 indicated that, except for the middle SDI and low SDI regions where ASPR and ASYR did not show significant declines, all other SDI regions exhibited significant downward trends in various ASRs (Table 1 and Supplemental Digital Content Figure S1, available at: http://links.lww.com/JS9/F264).

Among the 21 GBD regions, from 1990 to 2021, 16 regions showed significant declines in ASIR, 13 regions in ASPR, and 14 regions in ASYR. Notably, the ASIR in North Africa and the Middle East, as well as the ASPR and ASYR in the Caribbean, North Africa, and the Middle East, exhibited significant upward trends. The remaining GBD regions showed no significant changes in ASR. Eastern Sub-Saharan Africa experienced the most significant decline in ASIR, while high-income Asia Pacific saw the most notable decreases in ASPR and ASYR. Conversely, the ASIR in North Africa and the Middle East, along with the ASPR and ASYR in the Caribbean, rose significantly. In 2021, Australasia recorded the highest ASIR (232.18 per 100 000), ASPR (182.32 per 100 000), and ASYR (18.56 per 100 000), while Western Sub-Saharan Africa had the lowest ASIR (44.76 per 100 000), ASPR (22.60 per 100 000), and ASYR (2.35 per 100 000; Table 1 and Supplemental Digital Content Figure S1, available at: http://links.lww.com/JS9/F264).

**Figure 1.**
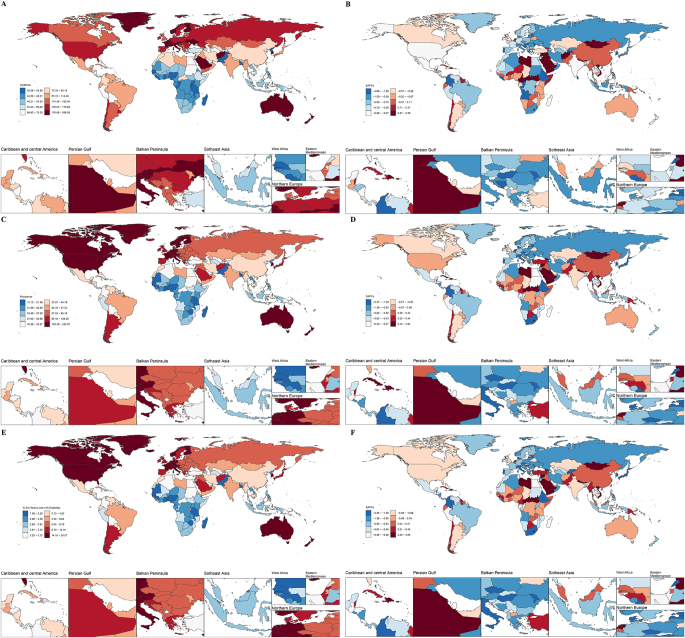
ASR and EAPC for 204 countries and regions in 2021 (A. ASIR; B. EAPC of ASIR; C. ASPR; D. EAPC of ASPR; E. ASYR; F. EAPC of ASYR).

ASR, age-standardized rate; EAPC, estimated annual percentage change; ASIR, age-standardized incidence rate; ASPR, age-standardized prevalence rate; ASYR, age-standardized rate years lived with disability.

Among the 204 countries and regions, the Andorra had the highest ASIR (264.30 per 100 000), ASPR (240.56 per 100 000), and ASYR (24.33 per 100 000) in 2021, followed by the Belgium and Finland. The Kiribati reported the lowest ASIR (30.87 per 100 000), ASPR (15.28 per 100 000), and ASYR (1.60 per 100, 000), followed by the Madagascar and Bangladesh (Fig. 1 and Supplemental Digital Content Table S1, available at: http://links.lww.com/JS9/F264). From 1990 to 2021, 125 countries showed significant declines in ASIR, 111 in ASPR, and 115 in ASYR. Conversely, 34 countries experienced significant increases in ASIR, while 45 countries saw significant rises in ASPR and ASYR. Notably, Syria exhibited the most substantial increases in ASIR (EAPC = 5.24), ASPR (EAPC = 4.79), and ASYR (EAPC = 4.81). Yemen (EAPC = 2.18) and Libya (EAPC = 1.90) followed as the second and third highest increases in ASIR, while Haiti (EAPC = 2.06/1.93) and Libya (EAPC = 1.63/1.60) ranked second and third for ASPR and ASYR, respectively. Among the countries showing a downward trend, East Timor (−4.61), Burundi (−4.59), and Liberia (−4.09) had the most significant reductions in ASIR. For ASPR and ASYR, Latvia (−2.35/–2.33), Taiwan (Province of China; −2.24/–2.27), and Lebanon (−2.06/–2.06) recorded the largest declines (Fig. 1 and Supplemental Digital Content Table S1, available at: http://links.lww.com/JS9/F264).

### Age-gender-time trend analysis

**Figure 2.**
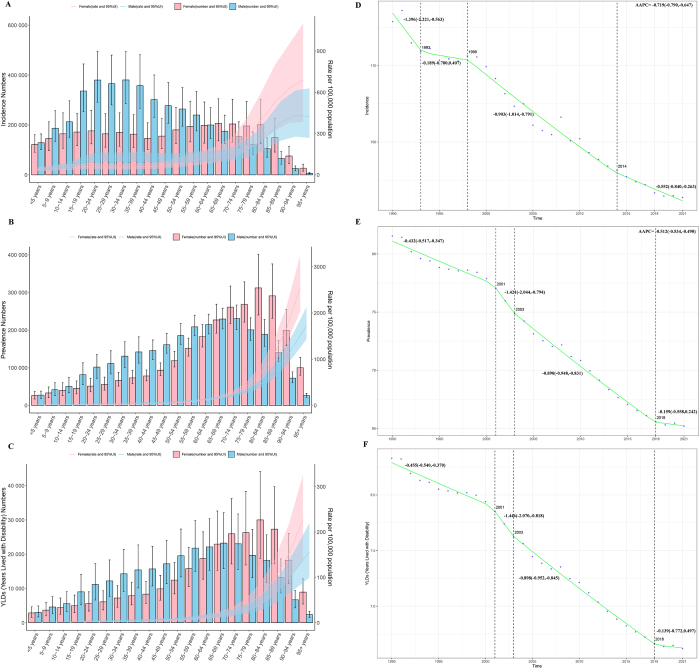
Age-gender trend and Joinpoint regression analysis results (A. ASIR; B. ASPR; C. ASYR; D. Joinpoint analysis of ASIR; E. Joinpoint analysis of ASPR; F. Joinpoint analysis of ASYR).

ASIR, age-standardized incidence rate; ASPR, age-standardized prevalence rate; ASYR, age-standardized rate years lived with disability.

The age-gender association analysis indicated that the ASR of spinal fractures increased with age. Before the age of 60, the ASR remained low, with males exhibiting higher ASR and actual case numbers than females. After age 60, the ASR rose significantly, with females surpassing males in both ASR and actual case numbers (Fig. 2A–C). Even after controlling for period and cohort effects, the analysis still demonstrated that the ASR of spinal fractures increased progressively with age for both males and females (Supplemental Digital Content Figure S2A–C, available at: http://links.lww.com/JS9/F264, Supplemental Digital Content Figure S3A–C, available at: http://links.lww.com/JS9/F264). The age-time association analysis revealed a downward trend in ASR across different age groups over time. However, the ASIR for individuals aged 90 and above showed a slow upward trend (Supplemental Digital Content Figure S4, available at: http://links.lww.com/JS9/F264). The gender-time association analysis indicated that both males and females exhibited a declining trend in ASR year by year, although there were noticeable increases in ASR for spinal fractures in 2004 and 2008 (Supplemental Digital Content Figure S5, available at: http://links.lww.com/JS9/F264). Similarly, after excluding the effects of age and cohort, the ASR for both genders continued to show a downward trend (Supplemental Digital Content Figure S2D–F, available at: http://links.lww.com/JS9/F264, Supplemental Digital Content Figure S3D–F, available at: http://links.lww.com/JS9/F264). Additionally, the period effect analysis indicated that later-born cohorts had lower ASRs for spinal fractures compared with earlier-born cohorts (Supplemental Digital Content Figure S2G–I, available at: http://links.lww.com/JS9/F264, Supplemental Digital Content Figure S3G–I, available at: http://links.lww.com/JS9/F264).

Joinpoint regression analysis results demonstrated a significant decline in all ASRs for spinal fractures from 1990 to 2021 (with AAPC and its 95% CI all being negative). Segmental analysis revealed significant turning points for ASIR in 1993, 1998, and 2014, with a consistent significant downward trend since 1998. ASPR and ASYR showed significant turning points in 2001, 2003, and 2018, but their downward trends have not been significant since 2018 (Fig. 2D–F).

### Correlation between ASR and SDI

**Figure 3.**
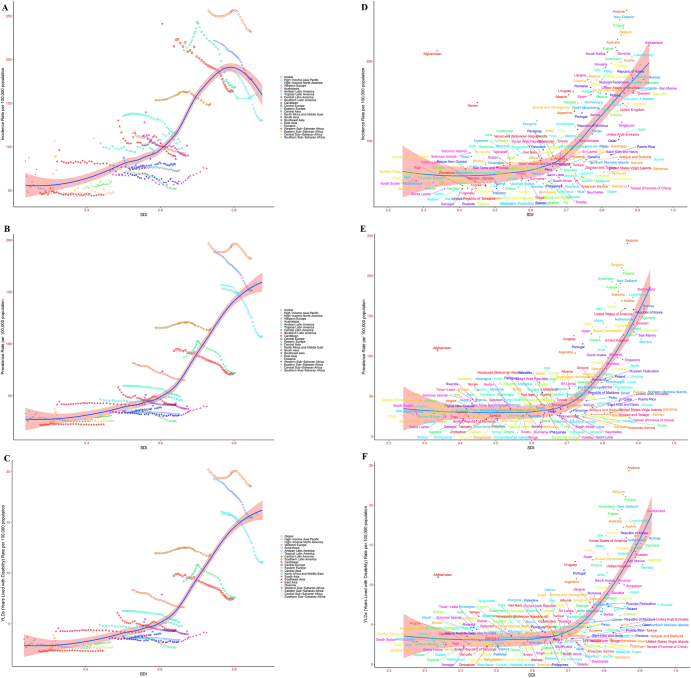
Correlation between ASR and SDI (A. ASIR; B. ASPR; C. ASYR).

ASR, age-standardized rate; ASIR, age-standardized incidence rate; ASPR, age-standardized prevalence rate; ASYR, age-standardized rate years lived with disability; SDI, Socio-Demographic Index.

Analysis based on the 21 GBD regions and 204 countries indicated that all types of ASR for spinal fractures significantly increased with rising SDI levels. Notably, when SDI reached 0.8, the ASIR began to decline, while other ASRs continued to rise (Fig. 3A–F). The correlation coefficients and *P*-values are presented in Supplemental Digital Content Table S2, available at: http://links.lww.com/JS9/F264.

**Figure 4.**
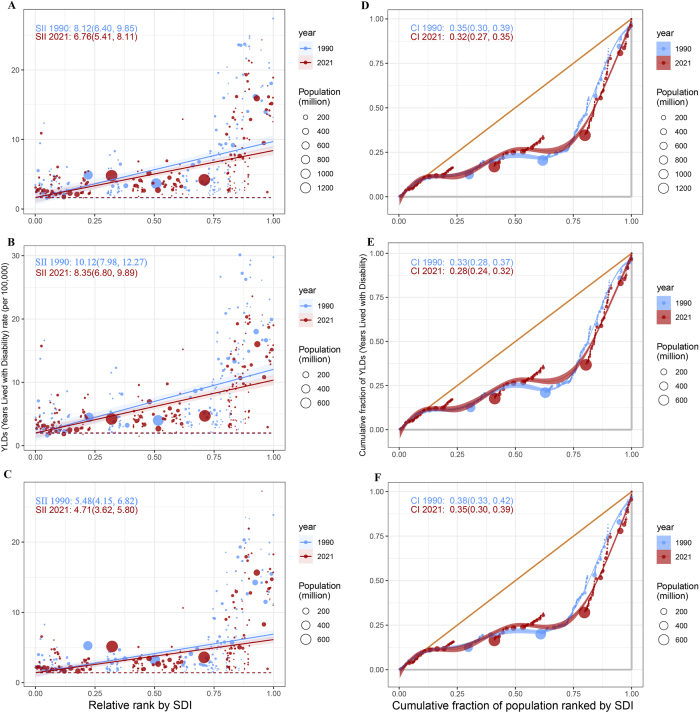
Health inequality analysis of YLDs (A. Overall SII; B. SII for males; C. SII for females; D. Overall CII; E. CII for males; F. CII for females). The size of the circles in the SII plots represents the population size, with larger circles indicating larger populations. The blue and red trend lines represent the relationship between YLDs and SDI rankings in 1990 and 2021, respectively. For the CII, the blue points correspond to the data from 1990, while the red points represent the data from 2021. The blue and red trend lines in the CII plots illustrate the relationship between cumulative YLDs and cumulative population scores in 1990 and 2021, respectively.).

CII, concentration index; SII, slope index of inequality; SDI, Socio-Demographic Index; YLD, years lived with disability.

The health inequality analysis based on YLDs reveals significant absolute and relative disparities among countries with varying Socio-Demographic Index (SDI) levels. The slope index indicates that from 1990 to 2021, the gap in ASYR between the highest and lowest SDI countries decreased from 8.12 (95% UI: 6.40, 9.85) per 100 000 years in 1990 to 6.76 (95% UI: 5.41, 8.11) per 100 000 years in 2021 (Fig. 4A). This suggests a reduction in absolute inequality, with the disease burden primarily concentrated in high SDI countries. Notably, the absolute inequality for men (Fig. 4B) is 43.58% higher than that for women (Fig. 4C). Additionally, the Concentration Index decreased from 0.35 (95% CI: 0.30, 0.39) in 1990 to 0.32 (95% CI: 0.27, 0.35) in 2021 (Fig. 4D), indicating an improvement in relative inequality between high-SDI and low-SDI countries, with the disease burden still primarily concentrated in high-SDI countries, and relative inequality being lower among males (Fig. 4E) compared with females (Fig. 4F).

Frontier analysis based on YLDs indicated that the ASR in most countries decreased continuously from 1990 to 2021 (Supplemental Digital Content Figure S6A, available at: http://links.lww.com/JS9/F264). The 15 countries and regions with the largest discrepancies from the optimal disease burden level (frontier benchmark) included Andorra, Belgium, Finland, New Zealand, Greenland, France, Switzerland, Australia, Luxembourg, Austria, the Republic of Korea, Norway, Italy, and the United States of America (with potential improvement ranges from 14.33 to 22.74). Among low-SDI countries (SDI < 0.50), those with the smallest differences from the frontier included Somalia, Niger, Malawi, Bangladesh, and Madagascar; whereas in high-SDI countries (SDI > 0.85), the countries with the largest differences from the frontier were Andorra, Belgium, Finland, Switzerland, and Luxembourg (Supplemental Digital Content Figure S6B, available at: http://links.lww.com/JS9/F264).

### Future predictions and causative analysis

It is projected that from 2022 to 2050, the ASR of spinal fractures will exhibit a marked downward trend (Supplemental Digital Content Figure S7A–C, available at: http://links.lww.com/JS9/F264), with the absolute number of patients significantly declining after peaking in 2022 (Supplemental Digital Content Figure S7D–F, available at: http://links.lww.com/JS9/F264). By 2050, the ASIR is expected to decrease to 60.55 per 100 000, the ASPR to 45.83 per 100 000, and the ASYR to 4.62 per 100 000 years (Supplemental Digital Content Table S3, available at: http://links.lww.com/JS9/F264). Furthermore, projections for different age groups indicate that, with the exception of the 90+ age group, where the ASIR is expected to remain relatively stable, the ASR is anticipated to decline significantly across all other age groups (Supplemental Digital Content Figure S8, available at: http://links.lww.com/JS9/F264). The absolute number of incident cases, prevalent cases, and YLDs is expected to decrease in age groups under 60, while showing an upward trend in age groups over 60. Notably, the increase becomes more pronounced with advancing age (Supplemental Digital Content Figure S9, available at: http://links.lww.com/JS9/F264).

**Figure 5.**
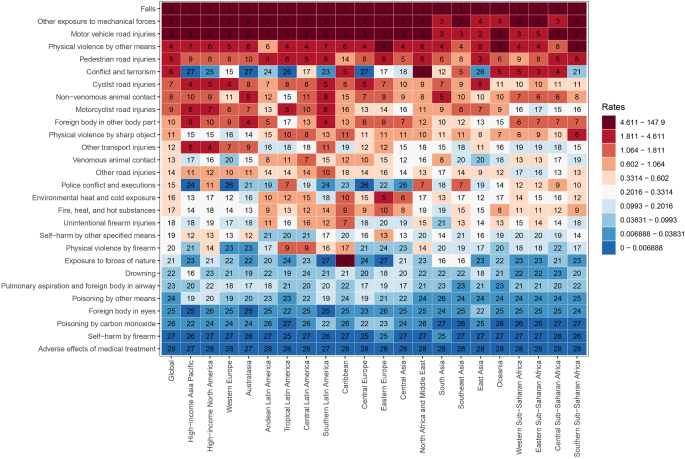
Causative factors for ASIR.

Currently, 28 causes of spinal fractures have been. At the global level and across the 21 GBD regions, the primary causes of spinal fractures are falls, other exposures to mechanical forces, and motor vehicle road injuries, while adverse effects of medical treatment, self-harm by firearm, and poisoning by carbon monoxide are the least common causes. Notably, in North Africa and the Middle East, as well as Eastern Sub-Saharan Africa, conflicts and terrorism rank as the third leading cause of spinal fractures (Fig. 5).

ASIR, age-standardized incidence rate.

## Discussion

This study provides a comprehensive quantification of the disease burden associated with spinal fractures. The results from the EAPC, AAPC, and age-period-cohort analyses indicate a significant decline in the disease burden of spinal fractures from 1990 to 2021, with an approximate reduction of 20%. Notably, the age-period-cohort analysis reveals an unusually high burden among individuals born between 1940 and 1950, likely related to the high exposure to trauma (such as combat injuries and heavy labor) and nutritional deficiencies experienced by this cohort during World War II and the post-war reconstruction period, which may have led to lifelong impairments in bone health. Predictions suggest a continued decline in the ASR of spinal fractures and a reduction in the overall absolute number of cases, affirming the effectiveness of public health interventions. However, age-stratified analyses reveal potential challenges, as the absolute number of cases in individuals over 60 is on the rise, with a more pronounced increase observed in older age groups. This trend is attributed to the interplay between population aging and conditions such as osteoporosis and multi-organ degeneration. Older patients not only face a significantly increased risk of fractures but also experience greater surgical complexity, longer rehabilitation periods, and higher resource consumption. Therefore, future healthcare systems must continue existing preventive strategies while focusing on developing screening and treatment frameworks for the elderly, such as community-oriented fall prevention programs and tiered rehabilitation approaches. Despite a significant decline in ASR in over half of the countries, there were still 7.4975 million new cases of spinal fractures reported in 2021. Notably, segmented regression analysis indicates that since 2018, the downward trend in ASPR and ASYR has begun to slow significantly, particularly in middle SDI and low SDI regions. In fact, regions such as North Africa and the Middle East, the Caribbean, and Oceania are experiencing a significant upward trend in ASR for spinal fractures. This underscores the need to timely adjust intervention strategies based on the latest disease burden data, especially in the 15 countries and regions with the highest burden, including Andorra, Belgium, Finland, New Zealand, and Greenland. Furthermore, an in-depth analysis of extreme trends reveals that Syria, Haiti, and Libya show the most pronounced increases in ASPR and ASYR, reflecting a vicious cycle of high-energy trauma and healthcare collapse due to conflict and disaster. Conversely, East Timor and Liberia have successfully reduced ASIR through international trauma initiatives, while Latvia and Taiwan (Province of China) have improved ASPR and ASYR through effective osteoporosis management. Lebanon has achieved a decrease in ASPR and ASYR despite being in a conflict zone, demonstrating the importance of targeted medical interventions. In summary, the spatial and temporal heterogeneity of disease burden provides critical insights for the allocation of healthcare resources and clinical service planning. In countries and regions where the disease burden remains high or is showing signs of increasing or slowing declines, healthcare systems and institutions need to enhance osteoporosis screening, promote key fall prevention measures, and initiate early anti-osteoporosis treatment for patients with fragility fractures to improve the severe burden of disease. At the same time, the overall declining trend in global burden affirms the effectiveness of existing treatment and prevention measures, encouraging clinicians to continue promoting evidence-based practices.

The study also identified a significant correlation between the ASR of spinal fractures and SDI, demonstrating that all types of ASR for spinal fractures significantly increase with higher SDI levels. This finding is supported by Spearman rank correlation analysis and health inequality analysis, which reveal significant absolute and relative inequalities between countries with different SDI levels, with the disease burden primarily concentrated in high-SDI countries. This is mainly due to the more advanced healthcare facilities and technologies in high SDI countries, leading to the identification and recording of more cases^[13]^. Furthermore, the lifestyles in high SDI countries tend to be more active, with greater participation in sports and high-risk activities, whereas people in low SDI countries, constrained by economic conditions, engage in fewer activities, reducing the incidence of spinal fractures. At the same time, high SDI countries face the challenges of an aging population, with older adults more prone to spinal fractures, particularly due to conditions such as osteoporosis^[18]^. However, in low SDI countries, the disease burden may be underestimated due to limited access to healthcare resources and higher rates of undiagnosed cases. In fact, the GBD study attempts to correct for diagnostic disparities by incorporating geospatial covariates and healthcare accessibility indicators. However, diagnostic biases between countries inevitably affect the disease burden estimates. It is noteworthy that as the economic level, medical conditions, and public awareness in high-SDI countries continue to improve, the incidence of spinal fractures in these countries (SDI > 0.8) has begun to show a downward trend. Our previous research has also demonstrated that the incidence of SCIs peaks when SDI reaches 0.8^[16]^, which aligns remarkably with the findings of this study regarding spinal fractures. This suggests that the formulation of public health policies should strictly consider the current economic development status of countries, and for traumatic SCIs, SDI = 0.8 is a critical threshold that warrants attention. It suggests that countries need to develop targeted prevention and treatment strategies based on their SDI levels. However, it is also important to note that in countries/regions where SDI is close to or exceeds 0.8, while the incidence of spinal fractures may plateau or begin to decline, the actual clinical service demand (such as surgeries, rehabilitation, and osteoporosis management) may remain high or continue to grow due to factors like population size, aging, and enhanced diagnostic capabilities. Therefore, corresponding clinical preparations should be made. Specifically, in resource-rich high SDI countries, efforts should focus on promoting vertebral augmentation surgeries (such as vertebroplasty and kyphoplasty) to effectively prevent the risk of re-fracture in patients with osteoporotic vertebral fractures. In resource-limited low SDI countries, priority should be given to implementing low-cost, high-impact interventions, such as community-based fall prevention programs^[19]^.

In-depth analysis of regional burden trends highlights the importance of identifying characteristic populations as a key step in formulating effective intervention measures. This study found that the ASR of spinal fractures increases with age. Before the age of 60, the disease burden remains relatively low. This is because spinal fractures are relatively rare among children and young adults, typically resulting from high-energy trauma such as motor vehicle collisions, ballistic injuries, or falls from heights^[20]^. This finding suggests that clinical practitioners should maintain a high index of suspicion for spinal fractures when managing trauma patients who are young males and optimize relevant imaging protocols. It also provides a targeted direction for clinicians to emphasize specific risk behaviors (such as protective measures for high-risk sports, traffic safety, and workplace safety) in patient education^[21]^. After the age of 60, the ASR of spinal fractures rises significantly, primarily related to osteoporosis associated with aging^[22]^. According to statistics from the United States, the incidence of spinal fractures due to osteoporosis is approximately 16%^[23]^. Osteoporosis leads to a significant reduction in bone density, thinning of trabecular and cortical bone, making the spine, as a major weight-bearing structure, particularly susceptible to fractures under osteoporotic conditions. Osteoporotic fractures have poor healing capabilities and are often accompanied by imbalances in bone remodeling, further increasing the risk of re-fracture^[2,24]^. Moreover, this study found that the ASR of spinal fractures in women aged 60 and older begins to exceed that of men, primarily driven by postmenopausal osteoporosis. Recent research has confirmed that the sharp decline in estrogen levels activates the RANKL pathway, enhancing osteoclast activity while inhibiting osteoblast differentiation, leading to rapid bone loss in women within 5–15 years post-menopause. This mechanism causes the degradation of trabecular microstructure in female vertebrae to occur at a much faster rate than in males, significantly increasing fracture risk^[18]^. Additionally, gender disparities in osteoporosis treatment rates exacerbate the gender heterogeneity of the burden of spinal fractures. The prevalence of osteoporosis in postmenopausal women is as high as 59.6%, yet less than half of this population (47.52%) receives treatment, primarily due to symptom attribution bias and limitations in medication accessibility^[25,26]^. Men face even greater challenges regarding the diagnosis, screening, and treatment of osteoporosis, which may lead to an underestimation of their disease burden^[27,28]^. It is noteworthy that approximately 15.81% of patients with thoracolumbar fractures also suffer from SCI, a severe comorbidity that significantly increases the risk of paralysis, neurogenic complications, and long-term care needs, greatly amplifying the overall disease burden of spinal fractures^[3]^. Previous studies on the global burden of SCI indicate that falls are the leading cause of SCI^[16]^. This finding aligns closely with our study’s identification of falls as the primary cause of spinal fractures, underscoring the importance of fall prevention in alleviating the burden of spinal and SCIs. In summary, this study provides an epidemiological basis for developing enhanced screening and prevention strategies targeting this high-risk population. It emphasizes the urgent need for systematic osteoporosis screening and proactive interventions (including bone density assessments, fall risk evaluations, nutritional supplementation, and anti-osteoporosis medication) for individuals over 60, particularly postmenopausal women, in primary healthcare settings.

Although the downward trend in the burden of spinal fractures is consistent across different age groups and genders, significant increases have been observed in certain regions or specific time periods, closely related to specific causes. For instance, the ASR for spinal fractures showed notable increases in 2004 and 2008, primarily due to the Indian Ocean earthquake on 26 December 2004, and the Wenchuan earthquake on 12 May 2008, which resulted in a large number of traumatic spinal fractures. North Africa and the Middle East, along with the Caribbean, are regions where the disease burden has risen most significantly, with conflict and terrorism, as well as physical violence by firearms, contributing substantially to this increase. With rapid societal development, spinal fractures resulting from traffic accidents and falls from heights have gradually increased. Research by Leucht *et al* indicated that falls from heights are the most common cause of traumatic spinal fractures, accounting for 39.56%, followed by injuries from car accidents at 26.5%^[9]^. This study also found that among the 28 causes analyzed, falls have surpassed motor vehicle road injuries to become the leading cause. Identifying these primary causes provides direct guidance for the formulation of clinical prevention strategies. As falls are the primary cause, particularly among the elderly, there is a strong case for promoting comprehensive fall prevention programs in clinical settings. These should include balance training, home environment assessments, vision checks, and medication reviews to reduce the use of drugs that may cause dizziness. For younger populations, prevention strategies targeting traffic accidents and falls from heights should emphasize safe driving education, the use of seat belts and helmets, and strict adherence to safety protocols for working at heights. Additionally, the observed increase in burden in specific regions, such as North Africa and the Middle East due to conflict, terrorism, and gun violence, underscores the need for clinicians to possess the necessary skills and vigilance when managing related injuries. It also calls for a concerted effort from the international community to address violent conflicts promptly. Notably, “other mechanical force exposure” (ICD-10: W20–W52), which ranks as the second leading cause of spinal fractures, encompasses occupational mechanical injuries, sports/recreational injuries, and unintentional daily exposures. This classification aligns with the World Health Organization’s International Classification of External Causes of Injury definition of mechanical injuries, emphasizing that prevention can be achieved through engineering controls (such as mechanical safety protections) and behavioral interventions^[29]^.

While this study provides a comprehensive and in-depth analysis of the disease burden of spinal fractures, some limitations must be acknowledged. First, the GBD data are sourced from a wide range of entities, including national health departments, international organizations, and medical institutions, leading to variability in data acquisition and quality across different locations^[14]^. Second, some patients may delay or be unable to seek treatment or may go undetected due to the absence of severe clinical symptoms, resulting in underreporting. Moreover, due to data availability constraints, the GBD database does not provide indicators that could be used to accurately quantify imaging diagnostic coverage at the national level (such as MRI/CT scanner density in specific years, accessibility to imaging in primary healthcare settings, or the diagnostic rate of spinal fractures by imaging). As a result, we are unable to directly isolate the impact of diagnostic bias on the correlation between SDI and the burden of spinal fractures through further sensitivity analysis (e.g., by including only countries with an imaging diagnostic rate >90%). This systematic disparity in diagnostic capacity is an important confounding factor when interpreting the global distribution of disease burden, particularly the finding that high-SDI countries bear a greater burden. Future studies should explore methods to integrate more detailed data on healthcare resources and diagnostic capacity to further calibrate burden assessments in regions with varying levels of development^[8,30]^. Third, the GBD data does not distinguish between specific spinal fracture segments (e.g., cervical, thoracic, and lumbar), while the risks of neurological injury, treatment complexity, and long-term prognosis vary significantly between fracture locations. This limitation restricts our ability to assess the impact of fracture site heterogeneity on disease burden^[31]^. Fourth, this study did not analyze differences in surgical rates across countries and their potential impact on YLD estimates. There may be substantial variations in surgical accessibility, the choice of surgical procedures, and post-operative rehabilitation resources between regions with different levels of development. These factors can significantly affect patients’ disability status and ultimately influence the YLD burden.

## Conclusion

From 1990 to 2021, the ASR of spinal fractures has significantly declined, and this trend is expected to continue. However, the absolute number of patients has gradually increased during this period. There are notable differences in disease burden between countries and regions, with the overall burden rising significantly alongside increases in the SDI, primarily concentrated in high-SDI countries. Additionally, the ASR increases with age, particularly showing a significant rise in individuals aged 60 and older, with females generally exhibiting higher ASRs than males. Falls, other exposures to mechanical forces, and motor vehicle road injuries are the primary causes of spinal fractures. This underscores the need for enhanced screening and treatment for osteoporosis, especially in high-burden areas and among the elderly population. In summary, the identification of high-risk populations, major preventable causes, and the spatiotemporal trends in disease burden revealed by this study provide critical evidence for clinicians to optimize screening strategies, strengthen preventive education, identify high-risk patients, and allocate resources effectively within healthcare systems.

## Data Availability

Data will be made available upon reasonable request.
